# Magnolol Loaded on Carboxymethyl Chitosan Particles Improved the Antimicrobial Resistance and Storability of Kiwifruits

**DOI:** 10.3390/foods12061149

**Published:** 2023-03-08

**Authors:** Feixu Mo, Wenzhi Li, Youhua Long, Rongyu Li, Yi Ding, Ming Li

**Affiliations:** 1 Institute of Crop Protection, Guizhou University, Guiyang 550025, China; 2 Research Center for Engineering Technology of Kiwifruit, Guizhou University, Guiyang 550025, China

**Keywords:** Magnolol@CMCS particles, foodborne pathogen, preservation coating agent, *Staphylococcus aureus*, kiwifruit

## Abstract

Magnolol is a natural compound extracted from the traditional Chinese medicine *Magnolia officinalis*, which exhibits antimicrobial properties. However, magnolol is insoluble in water and consists of a phenolic hydroxyl group, which is volatile; these factors hinder its application. In this study, a safe and environmentally friendly method to improve the microbial resistance and storability of harvested fruits is developed using the water-soluble carrier carboxymethyl chitosan (CMCS) and magnolol. Magnolol was loaded on CMCS particles to form Magnolol@CMCS antimicrobial particles, a preservation coating agent. Magnolol@CMCS particles effectively solved the problems of water insolubility and agglomeration of magnolol and reduced the size distribution D50 value of magnolol from 0.749 to 0.213 μm. Magnolol@CMCS particles showed greater toxicity against *Staphylococcus aureus*, *Escherichia coli*, and *Botryosphaeria dothidea* than that of magnolol alone, with effective medium concentration (EC_50_) values of 0.9408, 142.4144, and 8.8028 μg/mL, respectively. Kiwifruit treated with the Magnolol@CMCS solution showed delayed changes in fruit hardness and soluble solid and dry matter contents and significantly higher ascorbic acid (vitamin C) and soluble total sugar contents and sugar:acid ratios compared with that of the control fruit. In addition, no disease spots were observed on fruit treated with the Magnolol@CMCS solution within 7 days after inoculation with *B. dothidea*. In conclusion, Magnolol@CMCS particles showed antimicrobial activity on harvested fruits, effectively delayed the hardness and nutritional changes of fruits during storage, and improved the storability of kiwifruit.

## 1. Introduction

After harvesting, most fruits will transition from harvest to edible maturity through post-ripening, which is suitable for the duration of transportation and sales of fruits [1,2]. After the post-ripening process, the nutrients of the fruit are constantly consumed through a series of physiological functions and reactions, resulting in a notable decrease in nutritional and economic value [3]. Therefore, the time required for the post-ripening process is an important index for evaluating the storage performance of fruit. In addition, pathogenic microorganisms are important causes of damage to the value of fruits, including foodborne pathogens, such as *Staphylococcus aureus* and *Escherichia coli*, and pathogens of the fruit itself, such as *Penicillium* spp., *Botrytis cinerea*, and *Botryosphaeria dothidea* [4,5,6,7,8,9]. These pathogens can harm human health or damage the fruit.

Postharvest storage technology is key to maintaining the commercial and nutritional value of fruits. Currently, low-temperature storage is the most commonly used strategy; however, this approach cannot remove foodborne pathogens; *Penicillium* spp. and *B. cinerea* are still able to absorb fruit nutrients at low temperatures to maintain growth [10,11,12]. Therefore, dipping fruit in agentia has become an important measure to remove pathogenic microorganisms [13,14,15]. Dipping fruit in chemical pesticides can effectively remove pathogens during the storage period; however, non-standardized operations easily result in excessive pesticide residues on fruit, resulting in greater harm than benefit [16,17,18]. Therefore, dipping fruits in plant extracts with antimicrobial activities before storage is a safer alternative.

Magnolol is a natural compound extracted from the traditional Chinese medicine *Magnolia officinalis* [19]. As a polyphenolic compound, magnolol has been shown to have excellent antimicrobial properties [20]. Currently, studies have shown that magnolol exhibits obvious inhibitory effects on *Candida albicans*, *Acinetobacter baumannii*, *Helicobacter pylori*, and *S. aureus*; therefore, it is widely used as a medicine [21,22,23,24,25]. In addition, magnolol exhibits significant inhibitory effects on plant pathogens such as *Rhizoctonia solani*, *Alternaria alternata*, *B. cinerea*, and *P. expansum* [26,27,28,29]. Magnolol is a low-toxicity compound that has been proven harmless to goldfish [30,31]. However, magnolol is insoluble in water and consists of a phenolic hydroxyl group, which is volatile; these factors hinder its application [32,33]. Carboxymethyl chitosan (CMCS) is a chitosan derivative that can dissolve in water to form a viscous solution and has film-forming, antibacterial, hemostatic, and antioxidant properties. In addition, CMCS is a biocompatible and biodegradable drug carrier widely used as a medicine delivery technology [34,35,36,37,38].

In order to develop a safe preservation coating agent that can effectively improve the antimicrobial activity and the storability of kiwifruit, in this study, CMCS was loaded with magnolol to form Magnolol@CMCS antimicrobial particles with the aim of solving the problem of magnolol being insoluble in water and unstable. The Magnolol@CMCS particles were characterized using scanning electron microscopy (SEM), Fourier-transform infrared spectroscopy (FTIR), and thermogravimetric analysis (TG). The antimicrobial activity of Magnolol@CMCS particles against *S. aureus*, *E. coli*, and *B. dothidea* was determined. The changes in the storage properties of kiwifruit after Magnolol@CMCS solution treatment were determined. This study provides a new technical approach for improving the storage and preservation of fruit after harvest and food safety.

## 2. Materials and Methods

### 2.1. Materials

Chitosan (CS), CMCS, and hydroxypropyl-β-cyclodextrin (HP-β-CD) were purchased from Solarbio Technology Co., Ltd. (Beijing, China). Microsphere starch (MS) was provided by the Institute of Crop Protection, Guizhou University. Magnolol (≥98%) was purchased from Aladdin Biochemical Technology Co., Ltd. (Shanghai, China). *Escherichia coli* and *S. aureus* samples were provided by the Guizhou Institute of Light Industry (Guiyang, China). *Botryosphaeria dothidea* samples were provided by the Research Center for Engineering Technology of Kiwifruit, Guizhou University.

### 2.2. Preparation of Magnolol@CMCS Antimicrobial Particles

The preparation method of drug-loaded particles was based on the procedure of Hou et al. [39], and the influence of the controllable factors on drug loading was explored.

Evaluating the carriers: The four different carriers (CS, CMCS, HP-β-CD, and SM) were each added (2 g) into 50 mL distilled water and stirred for 1 h until completely dissolved. Magnolol (0.4 g) was dissolved in 5 mL methanol and added to 50 mL distilled water to form the magnolol solution. The magnolol solution was added into the different carrier solutions, and then, 10 mL was transferred into 50 mL centrifugal tubes for each solution. The tubes were oscillated at 1500 rpm for 6, 12, 24, 72, 120, and 168 h using an MS200 multi-tube vortex oscillator (Ruicheng Instrument Co., Ltd., Hangzhou, China). Then, the solutions were freeze-dried for 48 h. The dry substances were ground to obtain the drug-loaded particles. The drug-loaded particles (0.1 g) were added to 30 mL distilled water, stirred, and allowed to stand for 3 h. The supernatant was discarded, and 10 mL 98% methanol was added to dissolve the precipitate. High-performance liquid chromatography (HPLC) was performed to determine the content of magnolol in the precipitate, according to a protocol described by Zhou et al. [40]. HPLC was performed using a ZORBAX 300SB-C18 column with the measuring wavelength at 294 nm, mobile phase consisting of a methanol:water ratio of 78:22, sample size of 5 μL, and column temperature of 30 °C. Each treatment was repeated four times. The loading amount of magnolol in different loading particles was calculated using the equations below to identify the best carrier.

(1) $$\mathrm{Theoretical}\;\mathrm{drug}\;\mathrm{load}\;\left(\%\right)=\frac{\mathrm{Amount}\;\mathrm{of}\;\mathrm{magnolol}\;\mathrm{added}\;\left(g\right)}{\mathrm{Amount}\;\mathrm{of}\;\mathrm{magnolol}\;\mathrm{added}\;\left(g\right)+\mathrm{Amount}\;\mathrm{of}\;\mathrm{carboxymethyl}\;\mathrm{chitosan}\;\mathrm{added}\;\left(g\right)}\times 100$$

(2) $$\mathrm{Actual}\;\mathrm{drug}\;\mathrm{load}\;\left(\%\right)=\mathrm{Theoretical}\;\mathrm{drug}\;\mathrm{load}-\left(\frac{\mathrm{Amount}\;\mathrm{of}\;\mathrm{magnolol}\;\mathrm{in}\;\mathrm{the}\;\mathrm{precipitation}\;\left(g\right)}{\mathrm{Weight}\;\mathrm{of}\;\mathrm{the}\;\mathrm{drug}\;\left(g\right)-\mathrm{loading}\;\mathrm{particle}\;\left(g\right)}\right)\times 100$$

Evaluating the optimal loading ratio between CMCS and magnolol: The following weight ratios, 10:1, 5:1, 3:1, 2:1, 1:1, 1:2, 1:3, 1:5, and 1:10, were used to form 2% CMCS and magnolol mixed solutions, and then, 10 mL was transferred into 50 mL centrifugal tubes for each solution. The tubes were oscillated at 1500 rpm for 12 h using an MS200 multi-tube vortex oscillator. Then, the solutions were freeze-dried for 48 h. The content of magnolol in the particles was determined by HPLC. Each treatment was repeated four times, and the drug loading amount was calculated to obtain the optimal load ratio.

Evaluating the loading conditions: Three optimal proportions of CMCS and magnolol were selected for further study. The loading times were set to 6, 12, and 24 h, and the oscillation rates were set to 1000, 1500, and 2500 rpm. Through an orthogonal experimental design and according to the carrier screening method, different drug loading particles were prepared, and the content of magnolol in the particles was determined by HPLC. Each treatment was repeated four times, and the optimal loading conditions were obtained by calculating the amount of magnolol loaded. Finally, the preparation process of Magnolol@CMCS antimicrobial particles was established.

### 2.3. Characterization of Magnolol@CMCS Antimicrobial Particles

Magnolol@CMCS antimicrobial particles were characterized using the methods described by Liu et al. [38], with some modifications.

SEM observation: A small amount of magnolol and Magnolol@CMCS particles were dispersed in water and fixed onto the conductive adhesive. Then, the samples were sprayed for 45 s (gold spray, 10 mA) using the Oxford Quorum SC7620 sputter coater (Quorum Technologies Ltd., East Sussex, UK). The morphology of the samples was photographed using a ZEISS Sigma 300 scanning electron microscope (Carl Zeiss AG, Oberkochen, Germany).

Particle size analysis: CMCS was dissolved in water, magnolol was dissolved in 40% methanol, and Magnolol@CMCS particles were dissolved in water. The particle size distribution of CMCS and magnolol before and after loading was determined using an Omec LS-POP (9) laser particle size analyzer (Omec Instruments Co., Ltd., Zhuhai, China). The refractive indices of CMCS and magnolol were 1.648 and 1.602, respectively. Each particle was measured four times and averaged.

Surface area analysis: The pore and surface area characteristics of Magnolol@CMCS and CMCS were evaluated by measuring the nitrogen adsorption and desorption properties of the samples. The nitrogen adsorption/desorption test was conducted using a TriStar II 3020 surface area analyzer (Micromeritics Instrument Corp, Norcross, GA, USA), and the nitrogen adsorption and desorption curves of the samples were obtained. The Brunauer-Emmett-Teller method was used to calculate the surface areas and pore sizes of the samples. Each sample was measured four times and averaged.

TG analysis: The thermal stability of the samples was measured using a PerkinElmer TG-DSC analyzer (Waltham, MA, USA). Approximately 10 mg of each sample were added to a standard aluminum pot and heated from 30 to 600 °C at a heating rate of 10 °C/min and nitrogen flow rate of 100 mL/min to prevent thermal oxidation decomposition.

FTIR analysis: A Nicolet 670 infrared spectrometer (Thermo Fisher Scientific, Waltham, MA, USA) was used for the FTIR analysis. Magnolol, CMCS, magnolol and CMCS mixture, and Magnolol@CMCS particles were evaluated using the potassium bromide tablet test; 1 mg powder sample and 200 mg pure KBr were placed in the mold, pressed into a transparent sheet on the oil press, and the sample was evaluated using infrared spectrometry (wavenumber range, approximately 4000–400 cm^−1^; number of scans, 32; resolution, 4 cm^−1^).

### 2.4. Determination of the Antimicrobial Activity of Magnolol@CMCS Particles

The antimicrobial activity of Magnolol@CMCS particles was determined according to the methods described by Hao et al. [41] and Wu et al. [42], with minor modifications.

Determination of antibacterial activity: *Staphylococcus aureus* and *E. coli* were cultured in nutrient broth (NB) medium (5 g beef extract, 5 g NaCl, 10 g peptone, and 1000 mL distilled water, pH = 7.3) at 25 °C and 120 rpm/min for 24 h. Then, 1 mL bacterial solution was added into 150 mL nutrient agar (NA) medium (NB medium with 18 g agar) containing magnolol and Magnolol@CMCS particles of different concentrations. After shaking, 10 mL NA medium were transferred to a culture dish, allowed to cool and solidify, and incubated at 25 °C in the dark for 24 h. Different bacteria were cultured in NA medium without magnolol as the control. The number of colonies on the culture media was calculated, and the bacterial inhibition rates of the different concentrations of magnolol and Magnolol@CMCS particles were calculated. SPSS 20.0 (IBM, Armonk, NY, USA) was used to calculate the half-maximal effective concentration (EC_50_). In addition, continuously increasing the concentration of agents in NA until the minimum concentration that bacteria could not grow was determined, which was the minimum inhibitory concentration (MIC). Each treatment was repeated four times.

Determination of antifungal activity: *Botryosphaeria dothidea* was cultured on PDA medium at 28 °C for 4 days, until the colony diameter was 0.5 cm. PDA media containing different concentrations of magnolol and Magnolol@CMCS nanoparticles were inoculated using the *B. dothidea* culture and incubated at 28 °C in the dark; PDA medium without magnolol was used as the control. After 5 days, the colony diameter was measured, and the fungal inhibition rates of magnolol and Magnolol@CMCS nanoparticles with different concentrations were calculated using the equation below. Each treatment was repeated four times, and IBM SPSS Statistics 20.0 was used to calculate the EC_50_.

(3) $$\mathrm{Inhibition}\;\mathrm{rate}\;\left(\%\right)=\frac{\mathrm{Colony}\;\mathrm{number}\;(\mathrm{or}\;\mathrm{diameter})\;\mathrm{of}\mathrm{control}\;-\;\mathrm{Colony}\;\mathrm{number}\;(\mathrm{or}\;\mathrm{diameter})\;\mathrm{of}\;\mathrm{treatment}}{\mathrm{Colony}\;\mathrm{number}\;(\mathrm{or}\;\mathrm{diameter})\;\mathrm{of}\mathrm{control}}\times 100$$

### 2.5. Determination of Storability and Quality of Kiwifruit Treated with Magnolol@CMCS Particles

In August 2022, kiwifruit (*Actinidia chinensis* “Hongyang”) of similar sizes, produced in the same orchard, and harvested at the same time were randomly selected from 5-old-year kiwifruit trees. A total of 210 fruits were dipped in 3% Magnolol@CMCS solution, and 210 fruits were dipped in distilled water; these fruits were stored at room temperature (25 ± 2 °C). Thirty fruits were randomly selected at different times to determine the storability and quality. Kiwifruit storability and quality were determined using the methods described by Goldberg et al. [43] and Krupa et al. [44].

Determination of storability: The hardness of the equatorial part of the fruit was measured using a GY-4 fruit durometer (Handpi Instrument Co., Ltd., Leqing, China). The fruit was peeled and ground, the residue was filtered with gauze, and the soluble solid content in the filtrate was measured using an Abbe refractometer (YiCe Apparatus & Equipment Co., Ltd., Shanghai, China). After peeling the fruit, a transverse slice (thickness, 0.3 cm) from the equatorial part of the fruit was obtained, and its initial weight (m_0_) was measured; the slice was placed in an oven to dry at 80 °C until its weight was constant, and its weight (m_1_) was measured to calculate the dry matter content using the following equation:

(4) $$\mathrm{Dry}\;\mathrm{matter}\;\mathrm{content}\;\left(\%\right)=\frac{\;\left(m_{0}-\;m_{1}\right)}{m_{0}}\times 100$$

Determination of quality: The kiwifruit quality was determined using two treatments at a hardness of 1.0 kg/cm^2^. After the kiwifruit were peeled, 50 g were added to 50 mL 2% oxalic acid solution and homogenized; a 20 g homogenate was added to 10 mL xylene for extraction, and 15 mL of the lower layer solution was added to 1% oxalic acid solution for a final volume of 50 mL. Standard curves and regression equations were formulated using ascorbic acid standard solutions of 10, 50, 100, 250, and 500 μg/mL. The ascorbic acid solutions were quantified by HPLC using a C18 column with 0.1% oxalate solution as the mobile phase at the flow rate of 1.0 mL/min (wavelength, 254 nm; column temperature, 30 °C; sample size, 5 μL). The total soluble sugar was determined using a Handheld refractometer (Lichen-BX Instrument Technology Co., Ltd. Shanghai, China). The titratable acid content was determined via acid–base titration.

Resistance to fruit diseases during storage: Kiwifruit of similar sizes and without disease symptoms were selected, washed with distilled water three times, and dipped in either 3% Magnolol@CMCS solution (adding 3 g of Magnolol@CMCS into 97 mL water) or distilled water (control treatment). The fruits were allowed to stand for 1 h, the solution on the fruit surface was allowed to dry, and the surface of the fruit was inoculated with *B. dothidea* (colony diameter, 0.5 cm). The colonies were covered with the cotton moistened with distilled water to maintain moisture. The fruits were incubated at 28 °C with alternating light and dark, and the diameter of the lesion was measured 7 days later.

### 2.6. Statistical Analysis

All data were checked for normality and equality of variances prior to statistical analysis. All data were determined in quadruplicate, unless otherwise indicated, and the results were expressed as the mean ± SD (standard deviation). The loading ratio of different weight ratios of CMCS and magnolol, the loading ratio of different loading conditions, BET of different samples, D50 of different particles, and the quality index of kiwifruits by different treatments were analyzed using one-way analysis of variance (ANOVA), followed by Duncan’s multiple range tests (*p* < 0.05). All statistical analyses and graphs were performed by using IBM SPSS Statistics 20.0 and Origin 2021 (Origin Lab, Northampton, MA, USA), respectively.

## 3. Results

### 3.1. Magnolol@CMCS Antimicrobial Particles

**Figure 1:**
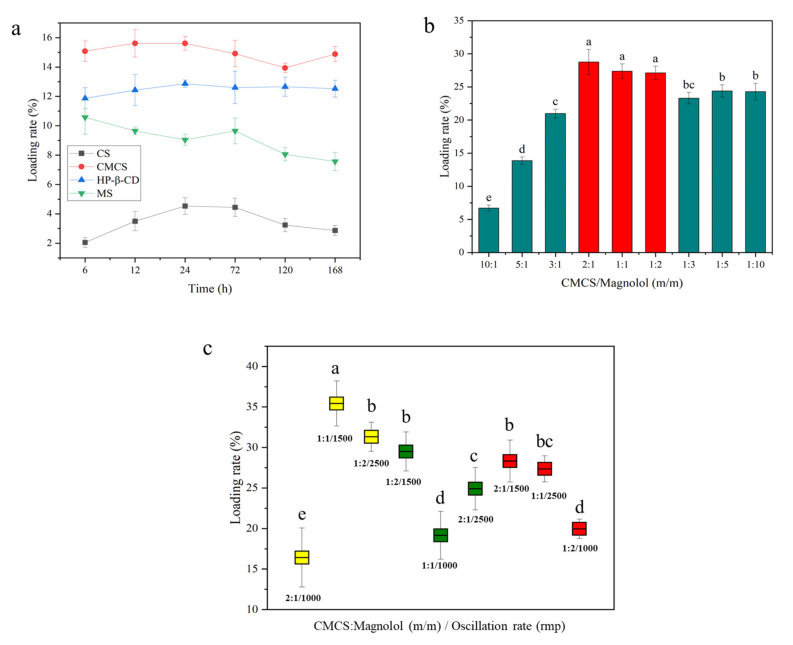
(**a**) The relationship between the amount of magnolol loaded on different carriers and time; (**b**) the amount of magnolol loaded on CMCS after different proportions of CMCS and magnolol oscillation (*n* = 4); and (**c**) magnolol load in CMCS after processing with different proportions, oscillation velocity, and oscillation time (*n* = 4). All data were expressed as the mean ± SD; the lowercase letters in the figure represent significant differences (*p* < 0.05).

Different carriers and magnolol were subjected to oscillating loads in the same proportion, and the load of magnolol from 6 to 168 h showed varying degrees of change (Figure 1a). The loading amount of magnolol on CMCS was higher than that on the other three carriers within 168 h; the drug loading rate of the particles was 15.62% at 12 h of oscillation. The drug loading rates of HP-β-CD and MS were 12.86% and 10.58% at 24 h of oscillation, respectively. The loading rate of magnolol on CS was <5% within 168 h, which was significantly lower than that of other carriers. CMCS was the most suitable carrier for loading magnolol.

The optimal loading ratio of CMCS and magnolol was explored using nine different weight ratios of CMCS and magnolol. The drug loading rate under the same loading condition is shown in Figure 1b. At the CMCS-to--magnolol ratios of 2:1, 1:1, and 1:2, the drug loading rate of the particles was 28.77%, 27.37%, and 27.12%, respectively, which were significantly higher than that of other treatments (*p* = 0.0049). Orthogonal tests were performed to further explore the optimal ratio, time, and oscillation rate of CMCS loaded with magnolol. The drug loading rate of the CMCS particles was 35.43%, with a CMCS-to-magnolol ratio of 1:1, oscillation rate of 1500 rpm, and oscillation time of 6 h, which were significantly higher than the other loading conditions (*p* = 0.0017) (Figure 1c). The resulting particles were Magnolol@CMCS antimicrobial particles.

### 3.2. Characterization of Magnolol@CMCS Antimicrobial Particles

**Figure 2:**
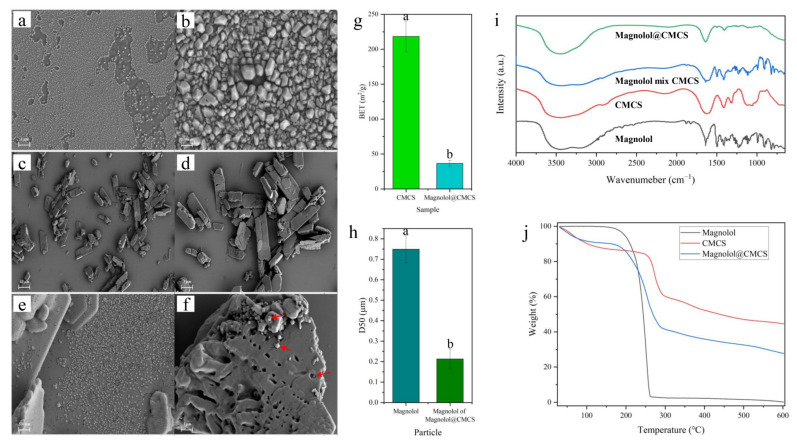
Scanning electron microscopy images of magnolol (**a**,**b**) and Magnolol@CMCS particles (**c**–**f**), the red arrows refer to magnolol; (**g**) the surface area of CMCS and Magnolol@CMCS particles (*n* = 4); (**h**) the particle size distribution of magnolol before and after loading (*n* = 4); (**i**) Fourier-infrared spectroscopy; and (**j**) thermogravimetric analysis. All data were expressed as the mean ± SD; the lowercase letters in the figure represent significant differences (*p* < 0.05).

**Figure 3:**
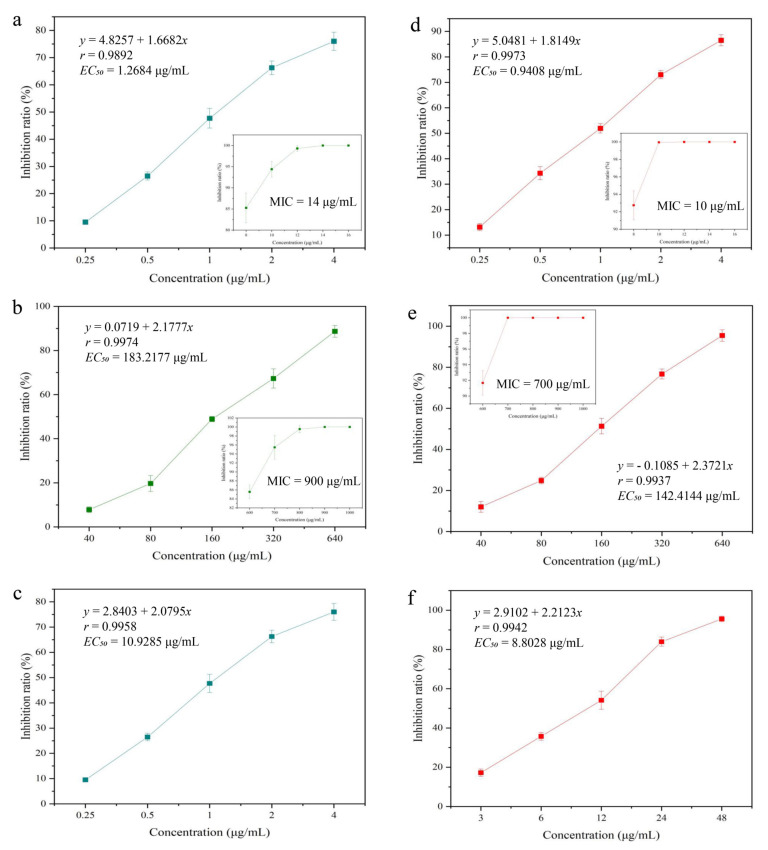
Toxicity of magnolol against *S. aureus*, *E. coli*, and *B. dothidea* (**a**–**c**). Toxicity of Magnolol@CMCS antimicrobial particles against *S. aureus*, *E. coli*, and *B. dothidea* (**d**–**f**).

SEM observation showed that, before loading, magnolol particles aggregated in water and were difficult to disperse (Figure 2a,b). This aggregation phenomenon would limit the permeability of magnolol, thereby limiting its antimicrobial effect. The Magnolol@CMCS particles formed by CMCS loading are shown in Figure 2c,d. Magnolol@CMCS presented irregularly shaped particles with different sizes. The Magnolol@CMCS particles exhibited large amounts of magnolol absorbed on the surface (Figure 2e) and in the gaps of the particles; large amounts of magnolol were also observed within the Magnolol@CMCS particles (Figure 3f). The specific surface area BET value of Magnolol@CMCS particles (36.61 m/g^2^) decreased significantly (*p* = 0.0015) compared to that of CMCS without loading magnolol (218.32 m/g^2^) (Figure 2g), which may be because the pore structure of CMCS particles was filled after loading magnolol. The results indicated that magnolol was successfully loaded into CMCS.

The magnolol particle size distribution D50 before loading was 0.749 μm, while the magnolol particle size D50 in Magnolol@CMCS was 0.213 μm (Figure 2h). The magnolol particle size was significantly reduced (*p* = 0.0062), which improved the antimicrobial activity. The FTIR analysis showed that the characteristic peaks of the magnolol and CMCS mixture were superpositions of the characteristic peaks of magnolol and CMCS (Figure 2i). Although the characteristic peaks of magnolol and CMCS were weaker in the characteristic front of Magnolol@CMCS, the corresponding characteristic peaks of the two substances could still be observed. The results showed that there was no chemical reaction between magnolol and CMCS in the Magnolol@CMCS particles; therefore, only physical adsorption was present. The TG analysis showed that the weight of magnolol began to decline when the temperature increased to 100 °C and approached zero when the temperature reached 600 °C, while the weight decline rate of Magnolol@CMCS particles was decreased, effectively improving the thermal stability of magnolol (Figure 2j).

### 3.3. Antimicrobial Activity of Magnolol@CMCS Particles

*Staphylococcus aureus* and *E. coli* are common foodborne Gram-positive and Gram-negative pathogenic bacteria, respectively (Figure 3a,b). Magnolol exhibits antibacterial activity against both *S. aureus* and *E. coli*. The EC_50_ and MIC values of magnolol against *S. aureus* were 1.2684 and 14 μg/mL, respectively. The EC_50_ and MIC values of magnolol against *E. coli* were 183.2177 and 900 μg/mL, respectively, indicating that magnolol was less toxic to Gram-negative bacteria. *Botryosphaeria dothidea* is a common fungal plant pathogen that can cause kiwifruit soft rot, resulting in fruit decay during storage and seriously damaging the quality and value of kiwifruit [45]. The EC_50_ of magnolol against *B. dothidea* was 10.9285 μg/mL (Figure 3c). After the formation of the Magnolol@CMCS particles, the toxicity against *S. aureus*, *E. coli*, and *B. dothidea* was evaluated (Figure 3d–f). The EC_50_ and MIC values for *S. aureus* were 0.9408 and 10 μg/mL. The EC_50_ and MIC values for *E. coli* were 142.4144 and 700 μg/mL, and the EC_50_ value of Magnolol@CMCS particles against *B. dothidea* was 8.8028 μg/mL.

The toxicity of magnolol against the three pathogens increased after the formation of the Magnolol@CMCS particles. This change may be associated with the change in water insolubility and aggregation of magnolol, resulting in increased contact between magnolol and pathogenic bacteria, thereby improving the antimicrobial activity.

### 3.4. Effects on Storability and Quality of Kiwifruit

**Figure 4:**
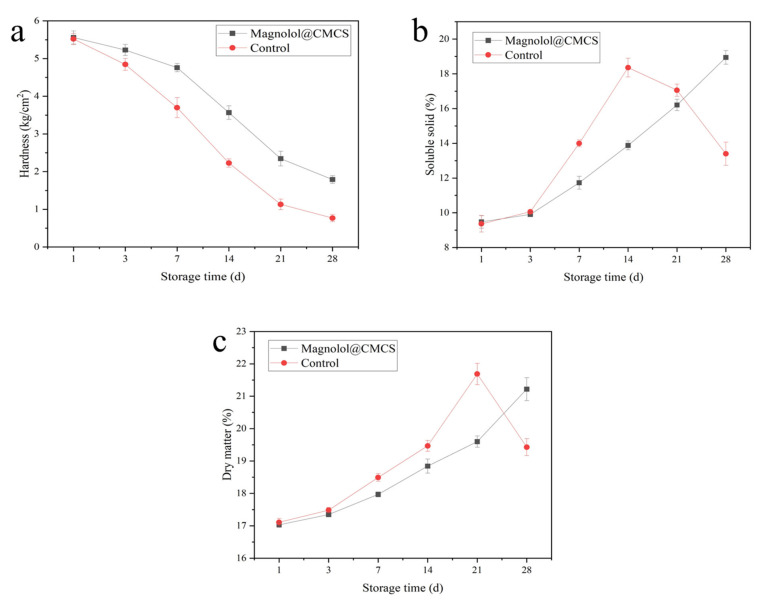
Changes of hardness (**a**), soluble solid content (**b**), and dry matter (**c**) of kiwifruit during storage under different treatments. Note: the kiwifruits were stored at room temperature.

The hardness of kiwifruit treated with Magnolol@CMCS solution started to decline after 7 days and was 1.79 kg/cm^2^ on the 28th day of storage, while the hardness of kiwifruit without Magnolol@CMCS solution treatment decreased rapidly during storage and was 0.77 kg/cm^2^ on the 28th day of storage, which was significantly lower than that of kiwifruit treated with Magnolol@CMCS solution (Figure 4a). The soluble solid content is an important index for evaluating the storability of fruit; the longer the time soluble solid content takes to peak during storage, the better the storability of the fruit [46]. The soluble solid content of kiwifruit treated with Magnolol@CMCS solution did not reach the peak value within 28 days of storage, while the control fruit reached the peak value and began to decline rapidly on the 14th day of storage (Figure 4b). During postharvest storage, the dry matter content of kiwifruit will increase due to water loss. After reaching the peak, the dry matter content of kiwifruit will decrease rapidly due to the fruit respiration. The longer the time for the dry matter content to peak, the better the storability of the fruit [47]. The dry matter content of kiwifruit treated with Magnolol@CMCS increased within the 28 days of storage without a peak value, while the dry matter content of the control kiwifruit reached a peak value on the 21st day of storage (Figure 4c). The Magnolol@CMCS solution delayed the decline of fruit hardness, prolonged the time to the peak soluble solid and dry matter contents during storage, and prolonged the storage time of kiwifruit.

The hardness of kiwifruit declines during storage; a hardness of 1.0 kg/cm^2^ is considered the most suitable for eating [48], and the quality of the fruit at this time is indicative of the nutrition and commodity values. The fruit hardness was approximately 1.0 kg/cm^2^ for both treatments, with no significant differences between the treatments (Table 1). At this time, the ascorbic acid and total soluble sugar contents of the kiwifruit treated with Magnolol@CMCS was 71.6200 mg/100 g and 14.3500%, respectively, which was significantly higher (*p* = 0.0071 and 0.0007) than that of the control fruit (66.3233 mg/100 g and 12.3400%). There was no significant difference in the titratable acid content between the two treatments; however, the sugar:acid ratio of kiwifruit treated with Magnolol@CMCS was 18.6274, which was significantly higher (*p* = 0.0048) than that of the control fruit (15.9255). This indicated that the kiwifruit treated with Magnolol@CMCS was sweeter than the control fruit. Magnolol@CMCS alleviated the quality changes of kiwifruit after harvest, delayed the loss of nutrients, and improved the commodity value.

**Figure 5:**
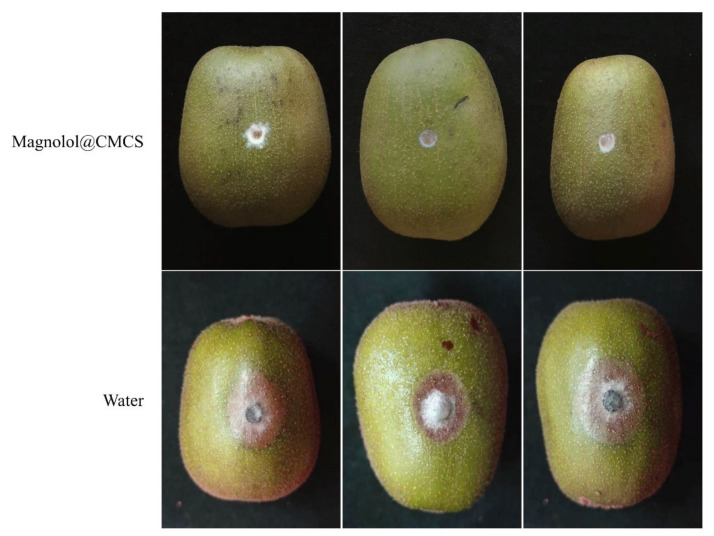
Effect of Magnolol@CMCS particles on preventing the fungal infection of kiwifruit. Note: the kiwifruits were inoculated with *B. dothidea* and then incubated at 28 °C for 7 d.

*Botryosphaeria dothidea* was used to inoculate the surface of kiwifruit treated with Magnolol@CMCS antimicrobial particles (Figure 5). After the seventh day, kiwifruit treated with Magnolol@CMCS antimicrobial particles exhibited mycelia that showed minimal growth on agar without infecting the kiwifruit and causing disease, and the fruit exhibited no disease spots. However, the control fruit exhibited obvious disease spots after inoculation with *B. dothidea*, mycelia invaded the inside of the fruit and caused fruit rot, and the average diameter of the disease spots was 2.14 cm. Magnolol@CMCS antimicrobial particles protected fruits from infection by pathogenic microorganisms during storage and had a significant application value for protecting fruit health.

## 4. Discussion

Magnolol is widely used as medicine, because it has been identified as having anticancer, anti-inflammatory, and antimicrobial effects; liver protection; and anti-mutagenesis properties [49,50,51,52]. As an extract of traditional Chinese medicine, magnolol is often used as an oral drug and has shown obvious positive effects on human health [53,54]. Therefore, the safety of magnolol is certain.

In this study, magnolol showed good inhibitory activity against Gram-negative bacteria, Gram-positive bacteria, and filamentous fungi. Chiu et al. showed that magnolol can reduce the formation of *S. aureus*, *S. mutans*, and *Aggregatibacter actinomycetemcomitans* biofilms, thereby inhibiting the growth of bacteria [55]. Mo et al. showed that magnolol destroys the integrity of the cytoplasmic membrane of the filamentous fungus *R. solani*, resulting in the inhibition of hyphal growth. In addition, magnolol exhibits antioxidant activity [26]. Lee et al. showed that magnolol scavenges free radical DPPH in vitro [56]. Chuang et al. showed that magnolol exhibits antioxidant effects by inhibiting the expression of inducible nitric oxide synthase and the production of nitric oxide and reactive oxygen species [57]. This may be the mechanism that results in improved fruit storability.

The antimicrobial and antioxidant properties of magnolol indicate that it can be used as a novel raw material for food packaging. However, there are few studies investigating magnolol as a food packaging material, as it is limited by the application technology of magnolol. CS, a polysaccharide with gel and film formation properties, is widely used in food packaging and has been shown to be safe for these purposes [58,59]. In addition, CS has antimicrobial properties, which can assist in preventing pathogenic microbial infection [60]. CS forms a protective barrier on the surfaces of fruits and aids in water retention and weight loss reduction, thereby improving fruit storage [61]. For instance, chitosan treatment could notably maintain the quality and increase the storability of longans during storage [62]. Chitosan at 5 g/L notably inhibited postharvest gray (*Botrytis cinerea*) and blue molds (*Penicillium expansum*) in kiwifruit [63]. However, compared with normal pesticides, the antimicrobial activities of chitosan and its derivatives are still not ideal [64]. Thus, researchers began to focus on the preparation of chitosan combined with pesticides or other active compounds. Song et al. developed a magnolol–chitosan film, which could significantly inhibit the growth of microorganisms in chilled pork and prolong its shelf life [65]. In another study, a SNP-incorporated chitosan film prepared by Shankar et al. exhibited strong antibacterial activity against *Escherichia coli* and *Listeria monocytogenes* [66]. As a derivative of CS, CMCS shows better water solubility. The Magnolol@CMCS antimicrobial particles developed in this study solved the problems of magnolol being almost insoluble in water and having poor stability and provided a fruit preservation coating agent, which allows the application of magnolol in food packaging.

## 5. Conclusions

The formulation and preparation of Magnolol@CMCS antimicrobial particles were as follows: the ratio of CMCS to magnolol was 1:1, the oscillation rate was 1500 rpm, and the oscillation time was 6 h. This resulted in a loading rate of magnolol in Magnolol@CMCS antimicrobial particles of 35.43%. Magnolol and CMCS exhibit antimicrobial properties; are nontoxic to humans; and exhibit significant inhibitory activities against *S. aureus*, *E. coli*, and *B. dothidea*. These properties protect fruits from infection by foodborne and plant pathogens during storage. In addition, Magnolol@CMCS antimicrobial particles delayed changes in the hardness, soluble solid and dry matter contents, and loss of nutrients and improved the storability and quality of fruits.

## Figures and Tables

**Table 1:** Quality of kiwifruit with different treatments in the appropriate edible period.

Treat	Hardness (kg/cm²)	Ascorbic Acid (mg/100 g)	Total Soluble Sugar (%)	Titratable Acid (%)	Sugar-Acid Ratio
Magnolol@CMCS	1.0383 ± 0.0743 ^a^	71.6200 ± 2.8476 ^a^	14.3500 ± 0.6162 ^a^	0.7767 ± 0.0449 ^a^	18.6274 ± 2.1538 ^a^
Control	1.0367 ± 0.0413 ^a^	66.3233 ± 2.4165 ^b^	12.3400 ± 1.1831 ^b^	0.7800 ± 0.0416 ^a^	15.9255 ± 2.0247 ^b^

Note: The values in the table are the mean ± SD; different lowercase letters indicate significant differences (*p* < 0.05).

## Data Availability

The data are available from the corresponding author.
